# Analysis and evaluation of explainable artificial intelligence on suicide risk assessment

**DOI:** 10.1038/s41598-024-53426-0

**Published:** 2024-03-14

**Authors:** Hao Tang, Aref Miri Rekavandi, Dharjinder Rooprai, Girish Dwivedi, Frank M. Sanfilippo, Farid Boussaid, Mohammed Bennamoun

**Affiliations:** 1 Department of Computer Science and Software Engineering, The University of Western Australia, Perth, Australia; 2 Armadale Mental Health Service, Perth, Australia; 3 Bethesda Clinic, Perth, Australia; 4 Advanced Clinical and Translational Cardiovascular Imaging, Harry Perkins Institute of Medical Research, The University of Western Australia, Perth, Australia; 5 Department of Cardiology, Fiona Stanley Hospital, Murdoch, WA Australia; 6 School of Population and Global Health, University of Western Australia, Perth, Australia; 7 Department of Electrical, Electronic and Computer Engineering, The University of Western Australia, Perth, Australia

**Keywords:** Computer science, Psychology, Human behaviour, Psychiatric disorders

## Abstract

This study explores the effectiveness of Explainable Artificial Intelligence (XAI) for predicting suicide risk from medical tabular data. Given the common challenge of limited datasets in health-related Machine Learning (ML) applications, we use data augmentation in tandem with ML to enhance the identification of individuals at high risk of suicide. We use SHapley Additive exPlanations (SHAP) for XAI and traditional correlation analysis to rank feature importance, pinpointing primary factors influencing suicide risk and preventive measures. Experimental results show the Random Forest (RF) model is excelling in accuracy, F1 score, and AUC (>97% across metrics). According to SHAP, anger issues, depression, and social isolation emerge as top predictors of suicide risk, while individuals with high incomes, esteemed professions, and higher education present the lowest risk. Our findings underscore the effectiveness of ML and XAI in suicide risk assessment, offering valuable insights for psychiatrists and facilitating informed clinical decisions.

## Introduction

Suicide accounted for $1.3\%$ of global deaths and was the $17\textrm{th}$ leading cause of death in 2019. With more than 700,000 individuals dying by suicide yearly, globally, the majority of deaths by suicide occurred in low-and-middle-income countries ($77\%$), where most of the world’s population live^1^. A survey in the United States also shows that $93\%$ of adults believe that suicides can be delayed or prevented if psychiatrists intervene effectively and immediately. According to a report published by the Centres for Disease Control and Prevention, middle-aged white men have the highest suicidal risk in the USA^2^, and suicide was the leading cause of death among Australian teenagers aged 15–24 in 2019–2021^3^. The current tools and solutions for suicide prevention mostly rely on self-reported measures, such as questionnaires and interviews, which can be subjective or multimodal data^4,5^ and is not easy to collect. Furthermore, traditional clinical risk assessment tools are not sufficiently accurate to identify individuals with moderate and high risk of suicide^6^. Two recent systematic reviews^7,8^ have evaluated various scales to predict the risk of suicide but have found overall low Positive Predictive Value (PPV). Hence, there is a critical need to develop technologies and models that can assist psychiatrists and mental health professionals to accurately stratify risk, enable precision medicine, and allocate resources.

Over the past decade, researchers have proposed various Machine Learning (ML) solutions and frameworks to enhance the performance of suicide prediction; however, since such models are primarily “black box” units and not interpretable, it is challenging to use them in clinical treatments. This study has three primary objectives. Initially, we conduct a comprehensive review of pertinent literature to collate and understand the various ML models employed for suicide prediction, placing an emphasis on their limited explicability in the context of clinical interventions. Subsequently, we embark on the selection and amalgamation of appropriate ML algorithms, leveraging data augmentation techniques to gauge the viability of ML models in predicting suicidal tendencies. Our final objective revolves around the identification of the most influential variables in suicide. To achieve this, we use an Explainable Artificial Intelligence (XAI) framework, which aids in discerning feature significance and offers a visual representation of the underlying reasoning behind the predictions.

The paper is organized in the following manner: “Literature review” Section provides a comprehensive literature review on the application of ML techniques in predicting suicide. Our findings, both visual and quantitative, are presented in Section “Results”. A more in-depth discussion and justification of the results are provided in Section “Discussion”. The methodologies used in this research are thoroughly explained in Section “Method”. Finally, the paper concludes with Section “Conclusion”.

## Literature review

ML is a branch of computer science that uses historical data to train models and make predictions about future trends. In recent years, there has been rapid growth and progress in the field of computer science, including ML, Computer Vision (CV), Artificial Intelligence (AI), and Natural Language Processing (NLP) which has led to the development of new tools and techniques to predict the risk of physical and psychological disorders^9^. For instance, these technologies have been implemented to predict the possibility of heart attacks^10^, lung and colon cancer detection^11^, liver diseases^12,13^, breast cancer detection^14^, brain analysis^15,16^, alcohol-related disorders^17^, human emotion disorders^18,19^, depression^20^, anomaly detection^21,22^, etc. In the past decade, studies have also shown that ML can be effective in predicting the risk of suicide^23,24^.

In recent years, numerous research studies have used ML techniques to predict suicide. For example^25^ integrated a C-Attention Network architecture with multiple ML models to identify individuals at risk of suicide. The three-stage suicide theory and prior emotions were also introduced to examine suicidal thoughts. In the sub-task of predicting suicide in 30 days, traditional ML models had superior performance compared to the baseline in the prediction, with an F1 score of 0.741 and an F2 score of 0.833 (higher F-score shows better performance^26^). Moreover, when predicting suicide within a 6-month period, the C-Attention method also outperformed the baseline, achieving an F1 score of 0.737 and an F2 score of 0.833. Other research has also utilized smartphone applications to gather data on participants’ therapy and apply NLP techniques to assess participants’ suicide risk levels^27^. The results showed that the Support Vector Machine (SVM) and Logistic Regression (LR) produced satisfactory calcification scores, while the extreme gradient model achieved the highest AUC value (0.78). The authors in^27^ highlighted the importance of using XAI tools to address the lack of explainability in traditional ML models, as it is crucial for psychiatrists to trust and rely on ML models. Similarly in^28^, the authors compared the performance of four traditional models, namely LR, Lasso, Ridge, and Random Forrest (RF), using the epidemiological Early Developmental Stages of Psychopathology (EDSP) dataset. After conducting nested 10-fold cross-validation, they found that these models performed almost the same in terms of mean AUC values ranging from 0.824 to 0.829. Furthermore, the RF model achieved the highest PPV of $87\%$, which was significantly better than other models. In suicide prediction tasks, various types of surveys, questionnaires, and scales have been used in the literature. For instance, in^29^ the Korea National Health & Nutrition Examination Survey (KNHANES) and the Synthetic Minority Over-sampling TEchnique (SMOTE) were used to select individuals with suicidal thoughts and to construct the dataset by resampling. After pre-processing, RF algorithm was applied and the experimental results verified the feasibility of such techniques on the general population. The RF model achieved an AUC of 0.947 and an accuracy of $88.9\%$. Notably, the feature selection process identified days of feeling unwell or in discomfort, daily smoking amount, and household composition as the most significant features that contributed to the prediction.

Traditional mathematical techniques produced less accurate results due to the complexity of input/output relationships in human behaviours. In^30^ the authors used the Participant Health Questionnaire-9 (PHQ-9) to collect data from college students and used the Mini-International Neuropsychiatric Interview suicidality module to evaluate their suicide ideation. They applied ML models, including K-Nearest Neighbourhood (KNN), Linear Discriminant Analysis (LDA), and RF. Their results showed that the RF model had the best performance, with an AUC of 0.841 and an accuracy of $94.3\%$. The positive and negative predictive values of the RF were also noteworthy, with values of $84.95\%$ and $95.54\%$, respectively. RF models were also used in other research studies, such as in^31^ to predict suicidal on a self-report dataset collected from 4,882 Chinese medical students. The dataset included clinical features from multiple psychiatric scales, including the Self-rating Anxiety Scale (SAS), the Self-rating Depression Scale (SDS), the Epworth Sleepiness Scale (ESS), the Self-Esteem Scale (SES), and the Chinese version of Connor Davidson Resilience Scale (CD-RISC). After applying five-fold cross-validation to the model, the experimental results showed that the RF model achieved significant performance, with an AUC value of 0.925 and an accuracy of $90.1\%$ in suicide prediction. This study also made several noteworthy discoveries, e.g., it found that relationships with caregivers were among the top five predictors of college students’ suicide risk prediction. ML algorithms have demonstrated potential in analyzing datasets from psychometric scales, such as the Suicide Crisis Inventory (SCI) and Columbia Suicide Severity Rating Scale (CSSRS)^32^. To improve model performance, researchers employed Gradient Boosting (GB) techniques to minimize prediction error and used SMOTE to generate artificial/synthetic data points. Their experimental results revealed that RF and GB algorithms performed the best, with precision values of $98.0\%$ and $94\%$ respectively for detecting short-term suicidal behaviours. An artificial neural network classifier with 31 psychiatric scales and 10 sociodemographic elements was proposed to predict suicide and assess the performance of ML models as well as identify the most significant variables^33^. The classifier’s accuracy for predicting suicide within a month, a year, and the whole lifetime were $93.7\%$, $90.8\%$, and $87.4\%$, respectively. In terms of the AUC, the highest was in 1-month detection (0.93), followed by lifetime prediction (0.89) and 1-year (0.87). In their study, the Emotion Regulation Questionnaire (ERQ) has the highest impact, followed by the Anger Rumination Scale (ARS) and the Satisfaction With Life Scale (SWLS)^33^. Recent studies have used various techniques to identify suicidal thoughts among certain populations. For instance, Haghish et al.^34^ used Machine Learning (ML) without relying on sensitive suicide-related survey questions. In^35^, ML methods were used to pinpoint students at risk of suicide during the COVID-19 pandemic. The work in^36^ focused on adolescents, assessing short-term suicidal risks post-hospitalization. An extensive review of these recent studies is available in^37^.

All the studies mentioned above were designed to apply standard ML techniques to predict the risk of suicide using their own private and imbalanced dataset with a small number of records. Additionally, to score the importance of each variable, conventional correlation analysis was commonly used. In contrast, in this current work, we not only use many ML algorithms for better comparison, but also employ data augmentation and state-of-the-art XAI frameworks to effectively analyze and interpret data.

## Results

**Figure 1:**
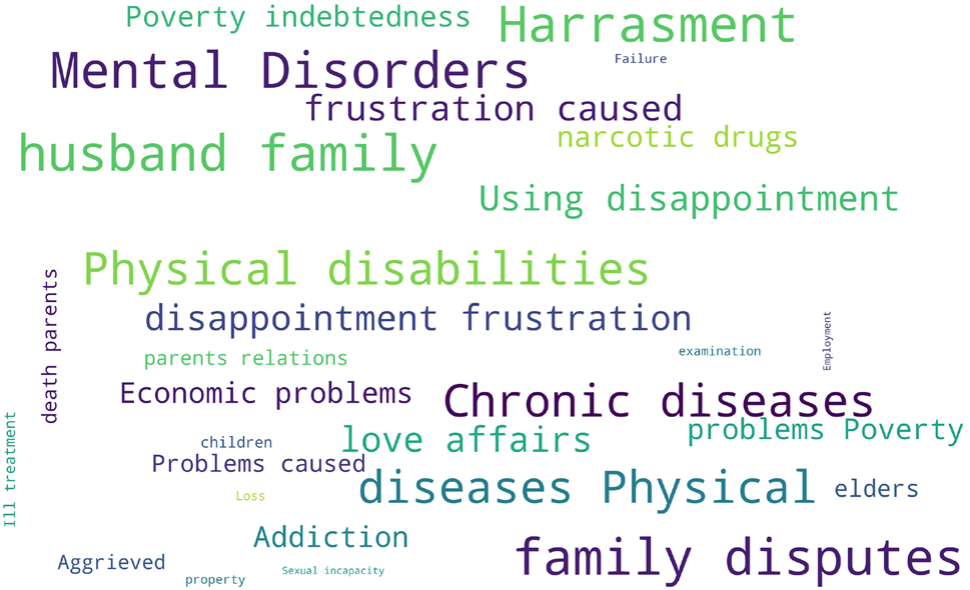
The word cloud of death reasons in the dataset. As shown, according to investigated data, physical and psychiatric disorders such as “physical disabilities,” “mental disorders,” “chronic diseases,” and “family disputes” are the most common reasons for death.

### Data visualization

**Figure 2:**
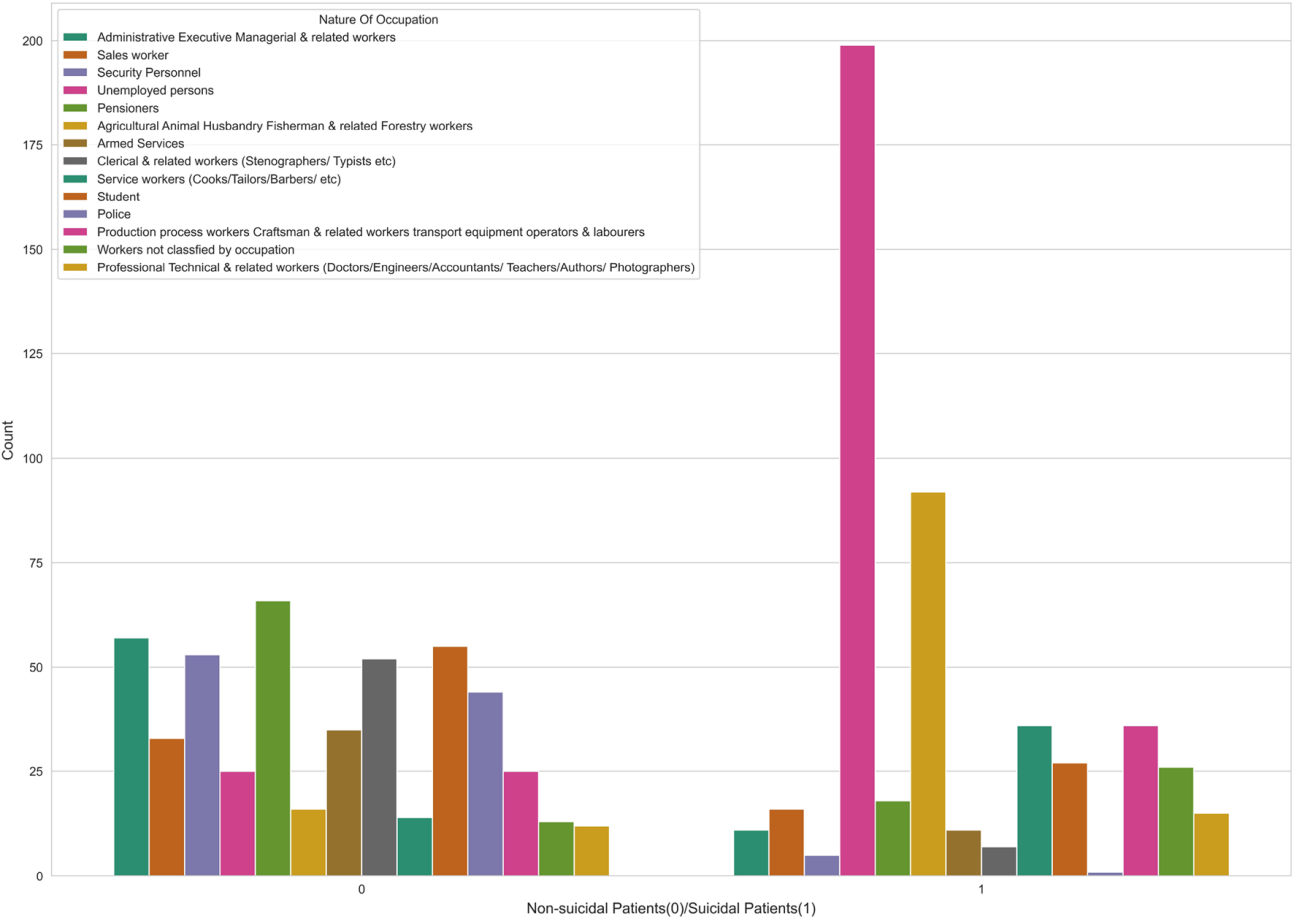
Counts of suicidal and non-suicidal records among different occupations. people who are Unemployed and people who work in agriculture and forest-related jobs are at a higher risk of suicide. On the other hand, police officers and security personnel are at the minimum risk.

The word cloud, a popular method in the NLP area, provides an intuitive illustration of the word frequency for people to understand which words appear most frequently in a given dataset. In the word cloud illustrated in Fig. 1, we observe that among the reasons for death, there are some high-frequency words, including “physical disabilities,” “mental disorders,” “chronic diseases,” and “family disputes.” We can assume that many people who died by suicide had physical and psychiatric disorders. Figure 2 on the other hand visualizes the count of people who died by suicide and people who did not, in each occupation. According to the used dataset, unemployment is a frequent attribute among suicidal behaviours, while people who work in agriculture and forest-related jobs are at a higher risk of suicide. An interesting discovery is that few police officers died by suicide, and the suicide rate of people with administrative management roles is relatively low among occupations. The conclusion of our visualization is similar to the results in previous studies^38^, which suggest that individuals with a good life income and people who can gain respect from their occupations are less prone to death by suicide.

**Table 1:** Models performances (in $\%$) and the standard deviation of the results on 100 trials of predicting suicide before (-b) and after (-a) data augmentation.

Models $\downarrow$**/Metric**$\rightarrow$	Accuracy-b	Accuracy-a	Precision-b	Precision-a	Recall-b	Recall-a	F1 Score-b	F1-Score-a	AUC-b	AUC-a
SVM	95.85±0.97	94.99±2.32	95.23±1.79	94.07±2.94	96.50±1.61	96.09±2.13	95.84±1.03	95.05±2.21	95.86±0.96	94.99±2.33
LR	96.13±1.02	95.86±0.99	95.56±1.67	95.00±1.69	96.74±1.67	96.88±1.55	96.13±1.04	95.91±0.96	92.62±1.40	93.31±1.36
DT	92.6±1.42	93.3±1.37	92.28±2.53	93.31±2.18	92.92±2.29	93.36±2.00	92.56±1.48	93.31±1.36	96.14±1.01	95.87±1.00
RF	96.80±0.80	**97.04**±**0.89**	95.30±1.72	**96.05**±**1.61**	**98.43**±**1.06**	98.15±1.15	96.82±0.82	**97.08**±**0.88**	96.82±0.79	**97.05**±**0.89**
Perceptron (iter=10)	91.98±6.07	94.70±1.91	93.35±6.28	93.72±3.84	91.32±12.73	96.12±4.19	91.45±7.77	94.77±1.96	92.01±5.97	94.72±1.90
XGBoost	94.65±1.36	94.86±1.34	93.62±2.41	94.74±2.01	95.79±1.91	95.05±1.87	94.66±1.38	94.88±1.34	94.68±1.35	94.88±1.32

Significant values are in bold

### ML performance

This section reports the performance of the implemented ML algorithms. We have implemented Random Forest (RF), Decision Tree (DT), Logistic Regression (LR), Support Vector Machine (SVM), Perceptron, and eXtreme Gradient Boosting (XGBoost) techniques to predict the risk of suicide based on records in the dataset. To minimize the potential effects of randomness on our experimental outcomes, we repeated the experiments 100 times, maintaining a consistent train-test split ratio of 70–30%. We then reported the mean values alongside their standard deviations to highlight the statistical performance of the models. These results are based on 19 numerical and categorical features present in the dataset. While certain machine learning models like RF and XGBoost inherently mitigate overfitting, there are other strategies we employ. These include synthetically expanding the dataset and closely observing the disparity between training and testing performances across various hyper-parameters, all aimed at reducing overfitting. Table 1 details the performance of each model, comparing various evaluation metrics for both the original and augmented version of the dataset. Based on the provided statistics, the RF model stands out with a commendable AUC value of 97.05%. Across all the evaluated metrics, RF consistently excels, surpassing the performance of the other machine learning models. Our simulations reveal that augmenting the data improved the outcomes in nearly all metrics, with precision being the sole exception. A notable performance boost across all metrics was observed due to the incorporation of the data augmentation technique (e.g., Accuracy improved from 96.80 to 97.04%, F1 Score from 96.82 to 97.08%, and AUC from 96.82 to 97.05%). To enhance the credibility of our models and understand the underlying reasons for their high performance, this research introduces a correlation matrix and XAI model to further investigate the reasons behind the above performances.

**Figure 3:**
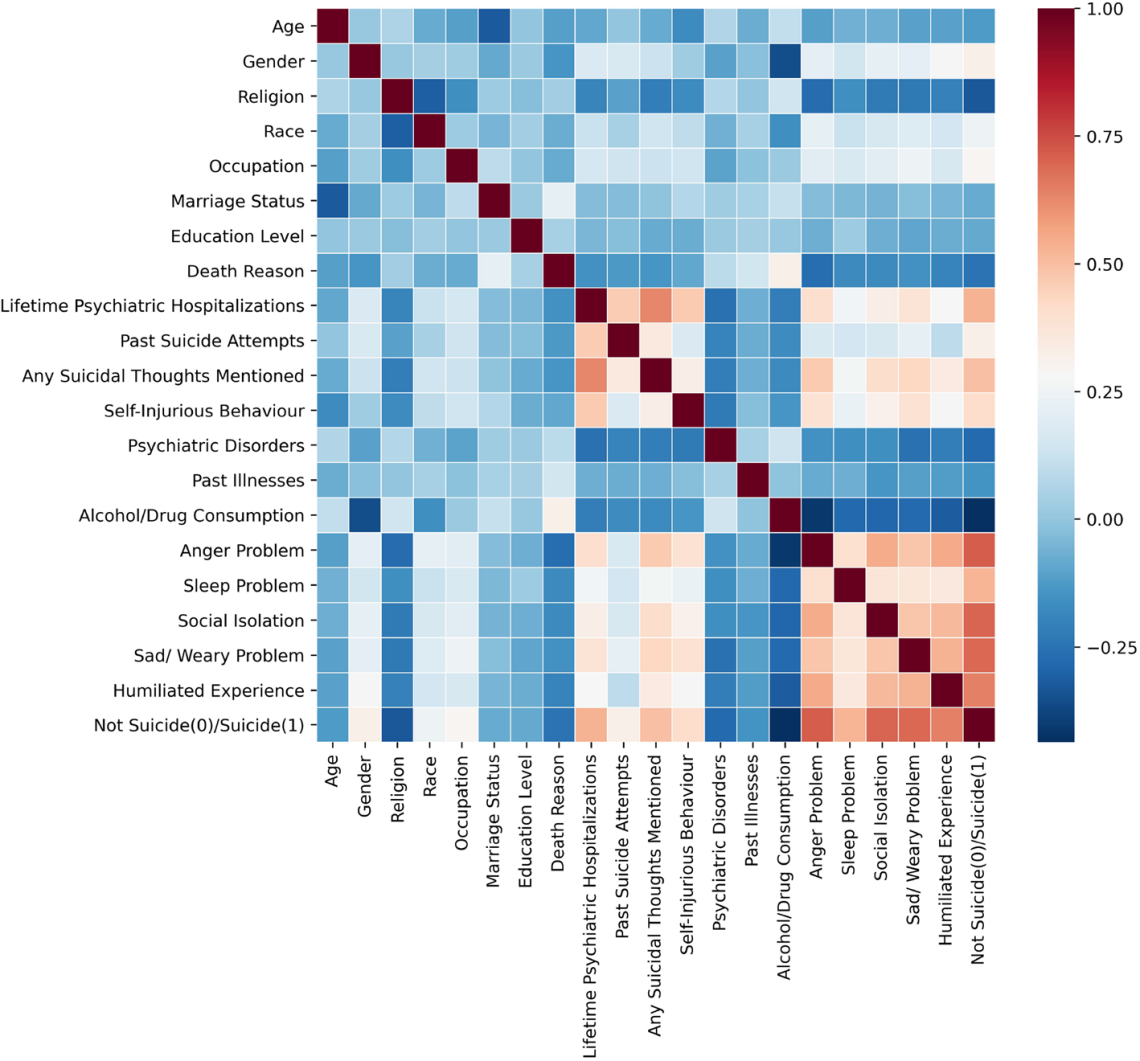
The correlation matrix of suicide-related variables. Results show a strong correlation between suicidal acts and anger problems, sleep problems, social isolation, depression problems, humiliating experiences, past suicide attempts, suicidal thoughts, self-injuries, and psychiatric disorders.

### Correlation analysis

In this section, we use the correlation function in Seaborn library and the heat-map function to analyze the correlation among attributes in the original dataset. Considering that many variables in the dataset are categorical and non-continuous variables, we use the Spearman correlation to perform the analysis. Figure 3 clearly illustrates the correlation between every two variables. According to the colour bar on the right-hand side, when the correlation colour between two variables is closer to 1, it is coded with dark red colour showing a significant positive correlation. A red area in the bottom right corner of the figure indicates that these variables are highly correlated. The heat-map shows a strong correlation between suicide and anger problems, sleep problems, social isolation, depression problems, and humiliating experiences. Moreover, the light red area in the center demonstrates a moderate correlation between the individual’s suicidal risk with past suicide attempts, suicidal thoughts, self-injuries, and psychiatric disorders. The above analysis proves that every single variable, which mostly measures mental disorders, can considerably contribute to the model prediction and the model would become more powerful when we combine all of these variables for prediction.

### Analysis by explainable AI

**Figure 4:**
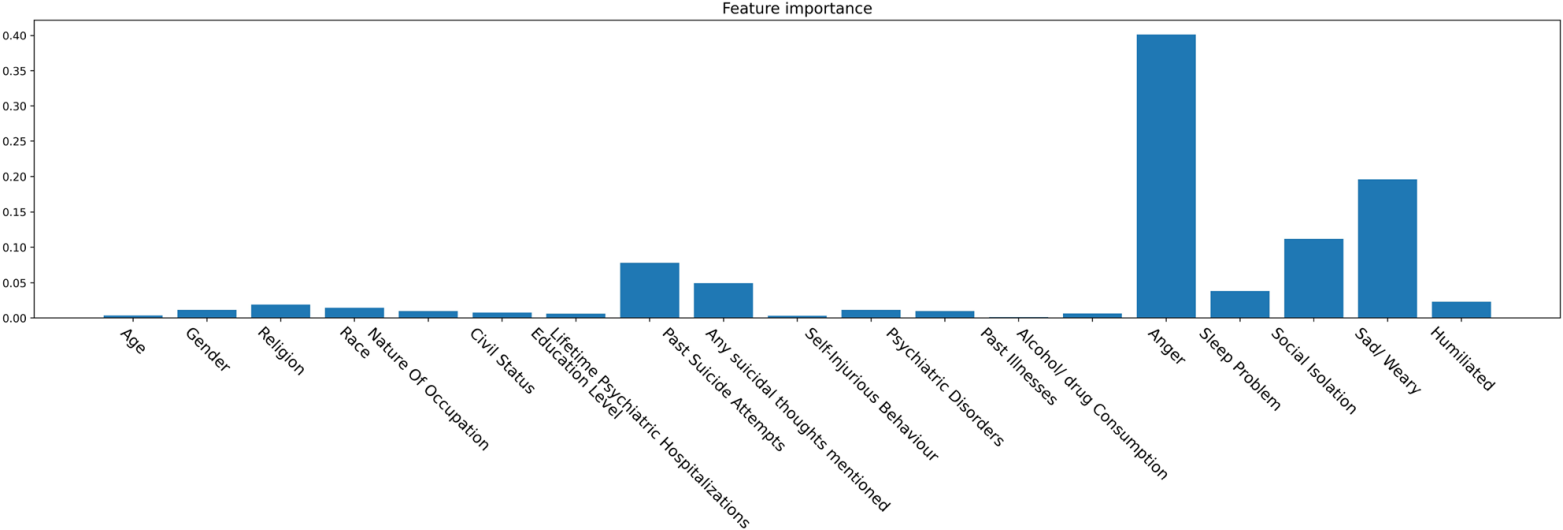
Traditional feature importance analysis provided by XGBoost predicting model. Similar to previous results, anger problem is the most important variable in suicidal risk prediction. The y-axis of the chart indicates the score of each feature with respect to other features in the dataset.

With the growing need to understand the underlying logic of ML models, studies have introduced the XAI framework to analyze the contribution of variables in model prediction. The generalization of SHapley Additive exPlanations (SHAP) and local interpretable model-agnostic explanation methods extend the use of XAI in the ML domain. Python package XGBoost provides functions to calculate the importance of features that contribute to the final model prediction. Figure 4 demonstrates such a feature’s importance in predicting suicide using XGBoost. It is evident that anger problem is the dominant variable correlated with suicidal behaviours. Mental health issues, including depression problems, social isolation, sleeping problem, and humiliating experiences are in next places and need psychiatrists’ attention. Meanwhile, past suicidal attempts and lifetime psychiatric hospitalization are important factors in detecting individuals who might die by suicide. Some nonlinear models, such as XGBoost, have strong prediction accuracy. However, their characteristics also make their interpretability inferior to linear models, which impedes them from being promoted in practical clinical diagnosis.

**Table 2:** SHAP value of a single sample.

Feature ID	Feature	Feature value	SHAP
0	Age	56	0.04823
1	Gender	1	0.0019
2	Religion	3	−0.03007
3	Race	1	−0.15723
4	Nature of occupation	5	−0.04402
5	Civil status	3	−0.05929
6	Education level	0	−0.00184
7	Lifetime psychiatric hospitalisations	0	−0.01955
8	Past suicide attempts	0	−0.00517
9	Any suicidal thoughts mentioned	0	−0.00878
10	Self-injurious behaviour	1	0.16583
11	Psychiatric disorders	3	−0.00353
12	Past illnesses	4	0.00875
13	Alcohol/ drug consumption	2	−0.00894
14	Anger	0	−0.11058
15	Sleep problem	1	0.01062
16	Social isolation	1	0.2493
17	Depression	0	−0.09246
18	Humiliated	0	−0.02779

**Figure 5:**
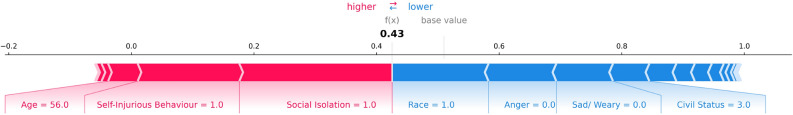
Variables with positive and negative contributions using SHAP analysis for a random participant. For this particular person, the model predicts that this person is at risk of suicide because of their age, past self-injury experiences, and social isolation.

In Table 2, we select a random sample from the original dataset and calculate the SHAP values for all 19 features involved in the prediction. SHAP value is a measure of feature importance in the model prediction based on a concept from cooperative game theory (see Section “Method” for more details). A large positive SHAP value indicates a strong direct contribution to the output prediction while a large negative value indicates the reverse effect in the predicted output value. Figure 5 illustrates the visualization of Table 2 and shows how these features compete and interact to end up with a decision. Features with red colour have positive contributions to the final SHAP value, while features with blue colour have negative contributions to the result. Our XGBoost model predicts that this person is at risk of suicide based on their age (56 years), past self-injury experience, and social isolation (which are the most significant positive factors for this person). Factors that make this decision uncertain include: not having an anger problem, no depression observed, being a Christian (the most important preventing factor for this person), being a widow, and being a clerical worker. The person is predicted to be at risk of suicide due to a stronger weight of positive factors relative to negative factors. Note that higher positive values in the output indicate a higher risk of death by suicide.

**Figure 6:**
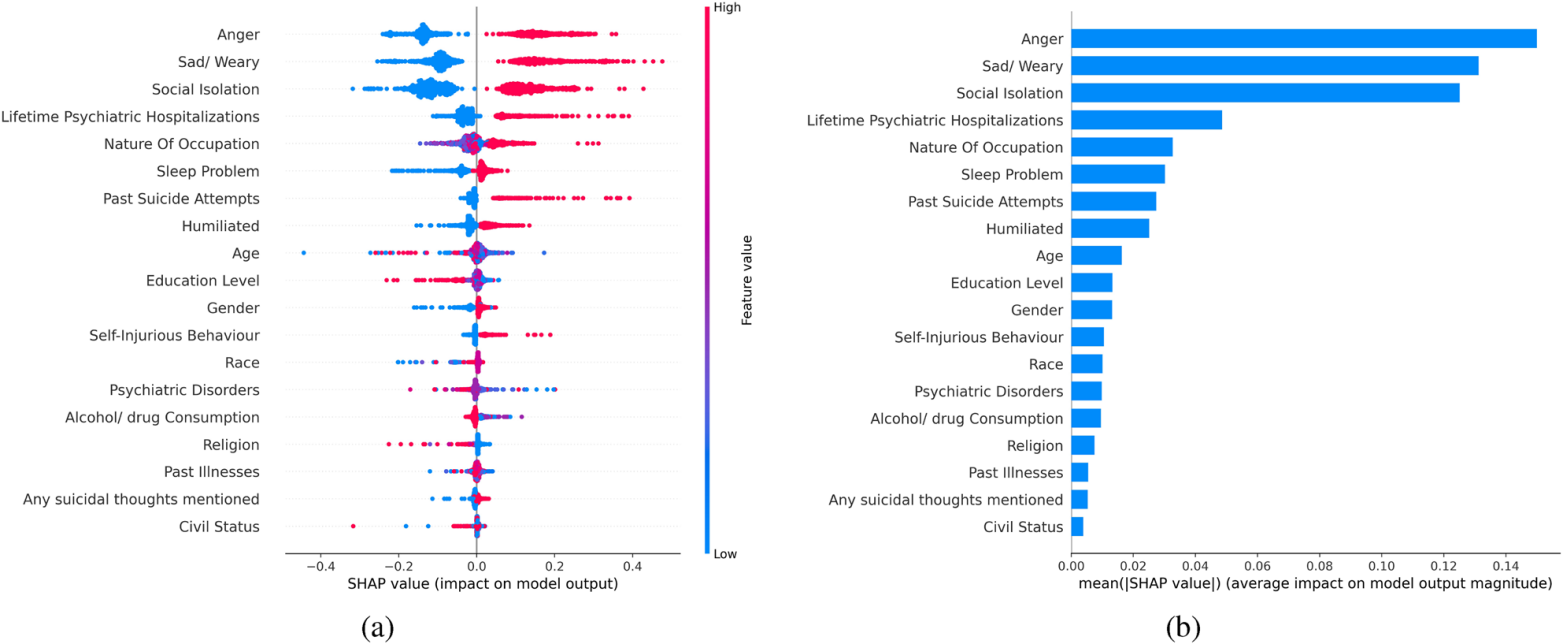
(**a**) Overall SHAP Values in the dataset. For each variable and participant, the contribution is shown by the SHAP value. A higher distinction between red and blue points shows higher importance in risk prediction, (**b**) Feature importance ranking of SHAP analysis. The top 3 variables are the same as the result shown in Fig. 4.

The SHAP method also provides interfaces to visualize the overall feature contributions. Figure 6a illustrates the overall SHAP value of features in our original dataset. Each individual is represented by a point. The red colour points indicate larger feature values, while the bluer colour points indicate lower feature values. It is noteworthy that for features such as past suicide attempts and self-injury behaviours, when the values of these features are low, people had a diverse experience and these variables do not negatively impact the predicted value. However, when the values of these features are high, indicating that these individuals have had suicidal attempts or self-injuries, these two features significantly contribute to a positive prediction.

**Figure 7:**
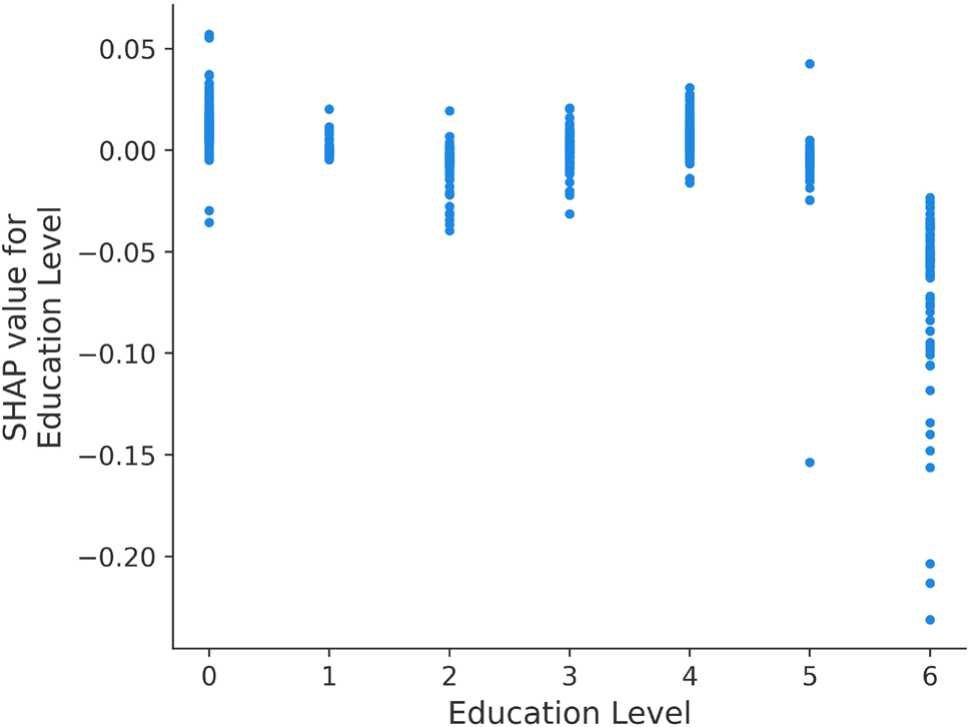
SHAP values for different educational levels.

Figure 6b shows the feature importance as calculated by the SHAP package. Although, there are some differences compared to Fig. 4, the top three variables, namely anger problem, depression problem, and social isolation remain the same. According to the importance rank provided by SHAP, psychiatric hospitalization, nature of occupation, and sleeping problems are also crucial variables in predicting suicide. To further analyze the impact of different feature values, the partial dependence plots from SHAP are used. For example in Fig. 7, each point represents one individual with a corresponding attribute value. It is observed that their distributions are closer to zero for most education levels and tend to be symmetric, indicating that this feature does not have a significant contribution to the final result. Figure 7 reveals that feature contributions are more pronounced for individuals with education level zero (from grade one to seven) and education level six (university degree or above). It can be observed that for most people with education level zero, their SHAP values are positive, indicating a higher risk of suicide, while all individuals with education level six have negative SHAP values, indicating a relatively low risk. This generally means that people with lower levels of education are at higher suicide risk, while those with university degrees are at lower risk.

### Clinical implications and future directions

Suicide prediction is difficult and traditional self-report-based risk assessment tools have been found to have limitations in predicting suicide. The other commonly used methods are clinical judgment and structured professional judgment. Clinical judgment alone has been found to have a sensitivity of $< 25\%$^39^. Most clinicians use structured professional judgment to determine risk. However, there is a need for tools or systems to validate the decision-making in suicide risk prediction. Carter et al.^7^ undertook a meta-analysis of three types of instruments used to predict death by suicide or self-harm: psychological scales, biological tests, and third-generation scales derived from statistical models. This review concluded that no instrument was sufficiently accurate to determine intervention. Similar to other areas of medicine, risk stratification is essential for accurate and precise treatment. The current paper has presented a methodology for improving suicide risk prediction using ML algorithms, which will hopefully increase the confidence of mental health professionals in utilizing ML algorithms in conjunction with clinical risk assessment to improve suicide risk prediction and intervention, and ultimately, to control the increasing rate of suicide worldwide. The next step of this study will be developing a risk assessment interface that utilizes the identified factors and ML algorithms to provide clinicians with a predicted measure of suicide risk for individuals. This will enhance and refine clinical decision-making. In future studies, it would be beneficial to investigate other modalities such as speech, image, and videos, as the current ML methods are often trained on text or tabular data.

## Discussion

This section justifies the excellent performance of ML algorithms in Table 1 by providing relevant evidence to support our experimental results. The dataset used in this study has a tabular format with both numerical and categorical attributes which are very common in medical applications. Standard and classical ML algorithms are particularly designed for such types of data under some mild assumptions such as uncorrelated features, a sufficient number of records, etc. DT is among these classical techniques which is specifically designed for tabular data and hand-crafted features. RF and XGBoost are advanced versions of DTs, which utilize the Bagging technique (ensembling technique) to combine the results of multiple trees resulting in improved predictive accuracy.

The superior performance of RF algorithm in the healthcare domain has been well-documented in several studies^40–42^. Research has found that models based on ensembling techniques significantly outperform other algorithms in predicting chronic stress and cardiovascular disease, with higher accuracy even when using fewer feature variables. The results and observations made in this paper align with the existing knowledge in psychiatry and provide a data-driven perspective to justify the experimental findings. The most important variables identified in this study can serve as a foundation for future research in the field.

The current paper aims to evaluate the performance of ML algorithms in predicting suicide and to improve the interpretability by using XAI framework. To achieve these objectives, the paper demonstrates an end-to-end process of using ML algorithms to predict suicide with XAI using tabular medical data. First, we conducted a literature review to summarise state-of-the-art suicidal datasets, psychometric questionnaires, ML models, and model evaluation parameters. Second, to prevent under-fitting when building models, the CTGAN and SMOTENC were used to generate a synthetic dataset without any data leakage. The CTGAN method had many powerful functions for data augmentation, but in terms of the distribution of feature values, the dataset generated by SMOTENC more closely resembles the distribution of the original dataset. In this paper, six models were built and repeated experiments were conducted to evaluate their performance. The Random Forrest (RF) showed excellent performance among the six models. Correlation analysis revealed that mental health disorders are strongly related to suicidal behaviours, which is consistent with existing research findings. Additionally, the XAI framework was applied to identify the dominant and key factors associated with suicide, which included anger problems, depression problems, social isolation, psychiatric hospitalization, and individuals’ occupation.

Our in-depth analysis revealed an enhancement in performance upon utilizing data augmentation, achieving over 97% accuracy in identifying people necessitating intensive care and additional examination. The findings underscored that certain features, like anger issues, depression, and social isolation, play a significant role in identifying suicidal tendencies irrespective of their values. Similarly, attributes such as a history of psychiatric hospitalizations, prior suicide attempts, and self-harm behaviors become crucial when they are registered with a “yes” response. Interestingly, individuals without sleeping disturbances tend to be at a reduced risk. Analyzing attribute quality, as depicted in Table 4, it is evident that most features exhibit clear value differentiation between groups. However, the average age presents an exception, with both groups displaying nearly identical values.

## Method

### Dataset selection

To determine the best dataset to use, we considered three criteria: (1) it should have a sufficient number of variables related to mental disorders, (2) the key outcome should be labeled, (3) it should be sufficiently large-scale to successfully apply ML techniques. In addition to these main objectives, the dataset variables should be easy to understand and should include textual variables, such as interview excerpts and symptom descriptions, to allow for the use of NLP techniques. The word cloud method developed by^43^ was used to effectively extract keywords from massive text information and intuitively visualize the importance of keywords. Based on the above criteria, we selected the anonymized dataset from a GitHub repository (https://github.com/dinisurunisal/Suicide-Risk-Prediction-Project/blob/master/DataScience/AlgorithmComparison/Test-Data-10.csv) for a software development project in the UK based on data collected from Colombo South Teaching Hospital-Kalubowila in Sri Lanka that is publicly available to everyone. The original dataset contained 22 variables, one of which was the output variable, and included 1000 records. This dataset was balanced, containing 500 positive and 500 negative samples. The age range of the participants spanned from 10 to 98 years. A concise overview reveals that this dataset includes 10 numerical attributes, with their respective statistics detailed in Table 3. These numerical attributes highlight the mental health challenges faced by the participants, capturing data on aspects such as lifetime psychiatric hospitalizations, past suicide attempts, suicidal thoughts, self-injuries, and more. In addition to these, the dataset also holds categorical variables offering holistic insights into the people’s sociodemographic profiles, including their occupation, marital status, and education level, all of which are valuable for the prediction task.

**Table 3:** Statistics for the original dataset (only numerical values).

Features $\downarrow$/Statistics$\rightarrow$	Mean	Std	Min	Max
Age	51.82	22.19	10	98
Lifetime psychiatric hospitalizations	0.23	0.42	0	1
Past suicide attempts	0.17	0.38	0	1
Suicide thoughts	0.31	0.46	0	1
Self-injuries	0.23	0.42	0	1
Anger problem	0.46	0.5	0	1
Sleeping problem	0.62	0.48	0	1
Social isolation	0.42	0.49	0	1
Depression problem	0.4	0.49	0	1
Humiliated experience	0.36	0.48	0	1

### Data preparation

**Figure 8:**
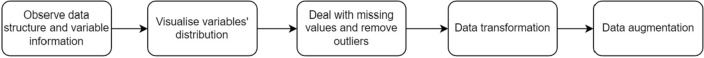
Data preparation steps.

The entirety of data tasks and subsequent analyses were conducted utilizing the Python programming language, complemented by its array of open-source libraries. The procedural steps involved in data preparation are depicted in Fig. 8, showing a representative data preparation pipeline. It is important to note that this dataset is free of missing values. For clarity and precision, we purposefully omitted the “Year” and “Reason” variables, deeming them either irrelevant or overlapping with the target variable. For consistency across all experiments, the dataset was partitioned into training and testing subsets with a 70%/30% ratio. Scikit-learn’s preprocessing library facilitated the encoding of categorical variables.

### Data augmentation

Most clinical datasets are of limited size and contain a small number of positive samples, leading to an imbalance. This often results in models that are biased and prone to overfitting. Although the dataset used for this study is balanced, it only includes 1000 records which is not sufficient to train a robust and high-performance model. To increase the dataset size, we implemented two data augmentation methods to enhance the dataset. One is the Conditional Generative Adversarial Network (CTGAN)^44^ and the other is the SMOTENC algorithm developed in Python for datasets with both numerical and categorical features.

The Tabular Generative Adversarial Network (TGAN) is the initial version of the CTGAN method. TGAN utilizes synthetic data generated by the conditional generative adversarial networks and has shown better performance than existing deep learning methods^45^. Moreover, CTGAN has several advanced functions, such as setting boundaries when generating numerical variables, conditional sampling, and creating primary keys for the dataset, which are added benefits. To achieve this augmentation, we used the default CTGAN model from the SDV library, focusing on the numerical features.

**Figure 9:**
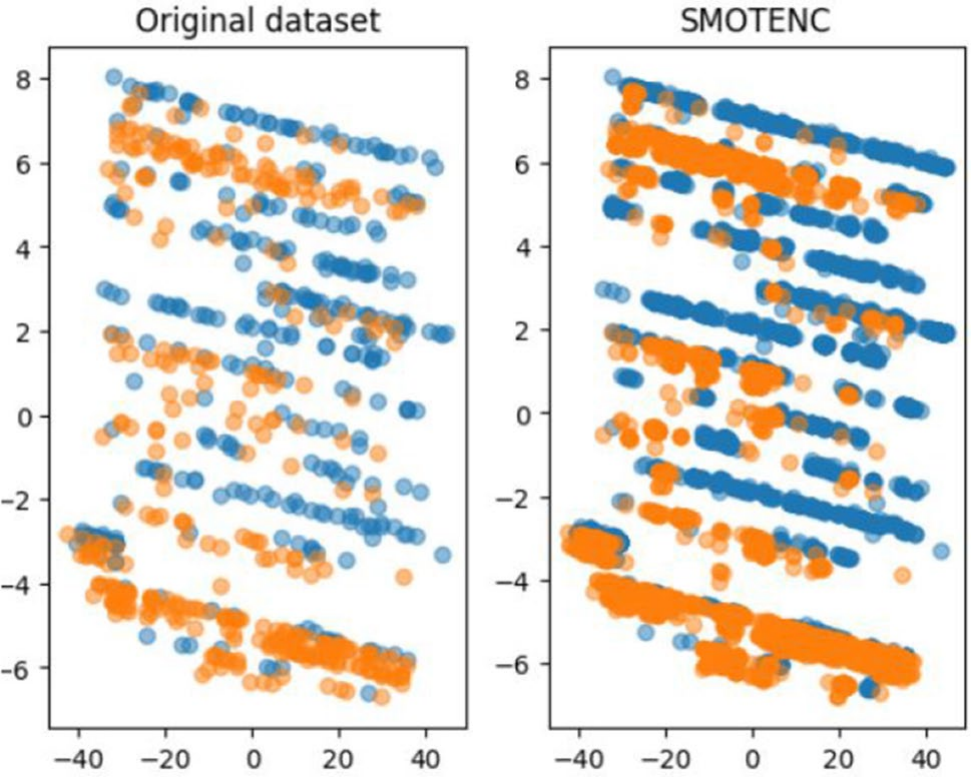
Principal component visualization of the original dataset versus augmented dataset using SMOTENC.

On the other hand, using SMOTENC we ensured that there is a similarity in the distributions between the original data and the data which is synthetically generated. For this specific data augmentation technique, we defined 9 features as categorical and 10 as numerical. Furthermore, we set the parameter for the number of neighbors to 5. Experiments were conducted to evaluate the performance of these two techniques on the selected dataset. We augmented the original training data to 10,000 samples and recorded the distributional information of each variable. Post-augmentation, we ensured the data set’s balance remained intact, with an equal distribution of 5000 samples for both positive and negative classes. Table 4 illustrates the first and second moments of variables’ distributions in the original and synthetic datasets. Among these variables, the statistics of SMOTENC-generated data are much closer to the original data, which indicates that the tools in SMOTENC maintain the original distribution better than CTGAN. Therefore, we decided to choose the synthetic dataset generated by SMOTENC as the augmented data for further steps. Figure 9 displays the two principal components of the augmented dataset alongside their original versions. As shown, the data distribution remains consistent, even though the data quantity has expanded.

**Table 4:** Mean and standard deviation of numerical features within classes, represented as (m, s), before and after data augmentation.

Dataset$\rightarrow$	Original		SMOTENC		CTGAN	
Features $\downarrow$/Group by$\rightarrow$	Suicide	Not suicide	Suicide	Not suicide	Suicide	Not suicide
Age	(49.01,20.98)	(54.63,23.01)	(49.07,21.03)	(55.25,23.01)	(59.21, 20.54)	(58.61,20.92)
Lifetime psychiatric hospitalizations	(0.45,0.49)	(0.01,0.49)	(0.46,0.46)	(0.005,0.06)	(0.11,0.31)	(0.12,0.32)
Past suicide attempts	(0.29,0.46)	(0.05,0.22)	(0.26,0.39)	(0.03,0.16)	(0.11,0.31)	(0.11,0.31)
Suicide thoughts	(0.54,0.5)	(0.08,0.27)	(0.52,0.45)	(0.05,0.19)	(0.07,0.25)	(0.06,0.23)
Self-injuries	(0.40,0.49)	(0.05,0.22)	(0.40,0.44)	(0.03,0.16)	(0.04,0.21)	(0.05,0.23)
Anger problem	(0.82,0.39)	(0.1,0.31)	(0.85,0.31)	(0.08,0.24)	(0.24,0.43)	(0.22,0.41)
Sleeping problem	(0.88,0.33)	(0.37,0.48)	(0.88,0.27)	(0.34,0.43)	(0.35,0.48)	(0.32,0.46)
Social isolation	(0.77,0.42)	(0.08,0.27)	(0.78,0.35)	(0.05,0.18)	(0.09,0.28)	(0.1,0.3)
Depression problem	(0.74,0.44)	(0.06,0.24)	(0.76,0.37)	(0.03,0.16)	(0.16,0.37)	(0.14,0.35)
Humiliated experience	(0.67,0.47)	(0.05,0.22)	(0.69,0.4)	(0.02,0.13)	(0.09,0.29)	(0.09,0.29)

Our primary objective in implementing data augmentation is to mitigate overfitting and improve generalization to the test set. However, an excessively large dataset size can lead to reduced model accuracy. Thus, we struck a balance by utilizing a sample size of 10,000.

### SHAP

The Shapley value, derived from cooperative game theory, offers a comprehensive method to evaluate the significance of every feature in a model prediction^46^. SHAP, an interpretative model based on the Shapley value, possesses several distinctive properties, including local accuracy, missingness, and consistency. Local accuracy ensures that the SHAP values offer a precise local interpretation of the model’s prediction for a specific input. Missingness implies that the SHAP value will be zero when an input is absent or when a feature is irrelevant. Consistency guarantees that the SHAP value remains consistent with any model alterations unless the contribution of a specific feature also changes. For each sample, the model generates a prediction value, with the SHAP value representing the value allocated to each feature within that sample^47^. For instance, if the $i\textrm{th}$ sample is denoted by $x_i$, the $j\textrm{th}$ feature of the $i\textrm{th}$ sample by $x_{i,j}$, the model prediction of the $i\textrm{th}$ sample by $y_i$ and the model’s baseline (typically the target variable’s mean value across all samples) by $y_{base}$, then the SHAP value is given by:

1 $$\begin{aligned} y_i=y_{base}+f(x_{i,1})+f(x_{i,2})+\cdots +f(x_{i,k}), \end{aligned}$$

where $f(x_{i,j})$ is the SHAP value of $x_{i,j}$, and *k* is the aggregate number of features. Intuitively, $f(x_{i,j})$ quantifies the influence of $x_{i,j}$ in determining $y_i$ and is ascertained through a combination of inclusion and exclusion of other features. When $f(x_i,j)>0$, it indicates that the feature positively impacts the prediction. Conversely, a negative value of $f(x_i,j)$ signifies that the feature diminishes the predicted outcome. Distinct from conventional feature importance methodologies, SHAP’s uniqueness lies in its ability to reflect the significance of feature values for individual samples, while also providing both positive and negative contributions of features. This facilitates attributing a contributory share to every feature. Features with high positive or negative SHAP values exhibit strong direct or inverse relationships with the predicted outcome, respectively. In the context of our study, this reveals which variables act as drivers for suicide and which ones act as deterrents. Conversely, SHAP values near zero underscore the feature’s lack of relevance to the output, as highlighted by the missingness property.

### Machine learning models

In our study, we used several classical machine learning models, including LR, DT, RF, SVM, Perceptron, and XGBoost. This section offers a concise overview of the foundational principles behind each model and the hyperparameters used for each model:

- *Logistic regression (LR) * One of the pioneering classification techniques, LR was primarily formulated for binary classification tasks. It seeks to maximize the likelihood of observed labels concerning the parameters of a linear model channeled through a nonlinear function, such as the Sigmoid function. Our implementation used the default LR model with an $\ell _2$ penalty, a stopping tolerance of $10^{-4}$, the “lbfgs” optimizer, and a regularization strength of 1.
- *Decision tree (DT) * DT is a classification method that uses a tree-like structure. It begins with a root feature and splits the feature space to achieve maximum label purity, using metrics like the Gini Index or Entropy. Our DT model was implemented using the Gini index as the criterion and the “best” splitter, necessitating a minimum of 2 samples to split an internal node.
- *Random forest (RF) * RF addresses the limitations inherent to DT by deploying multiple DTs, each trained on distinct feature subsets. This approach considerably diminishes overfitting and offers superior generalization to unseen data^48^. Our RF model was constructed with 100 estimators, and the DT-related parameters mirrored those in our DT model.
- *Support vector machine (SVM)* SVM is a classification methodology centered around discerning the optimal hyperplanes that segregate data points of two classes, focusing predominantly on boundary points and leveraging non-linear strategies. Our SVM implementation utilized a linear kernel with a cap of 5000 iterations.
- *Perceptron * The Perceptron is a supervised machine learning algorithm designed to determine a decision boundary between two data point clusters. It achieves this using the backpropagation method. For our analysis, we used the standard model provided in Scikit-learn, setting it to a maximum of 100 iterations, with $\alpha =10^{-4}$, and an elastic net mixing parameter of 0.15.
- *XGBoost * XGBoost operates on the principle of gradient-boosted decision trees, whereby multiple weak learners are aggregated to formulate a strong decision-making model. For our implementation, we opted for the default settings: the “gbtree” booster, a step size shrinkage of 0.3, a maximum depth of 6, and a uniform sampler.

### Ethical approval

This project was approved by the Royal Perth Hospital Human Research Ethics Committee (Approval Number RGS 4360). No personal data was processed in this study and the dataset used for this study is a publicly available anonymized dataset.

## Conclusion

Notably, this study is among the pioneering efforts that have harnessed Explainable Artificial Intelligence (XAI) tools, specifically SHapley Additive exPlanations (SHAP), to pinpoint the most critical factors contributing to suicide. A significant revelation from our analysis is that anger problems emerged as the primary cause of death by suicide based on the dataset under examination. It was shown that Machine Learning (ML) methodologies, when combined with augmented datasets, offer significant support to psychiatrists in identifying people at risk and reducing fatalities associated with mental disorders. The achieved performance underscores the potential of AI in enhancing clinical mental health diagnostics. For enhanced reliability and robustness of these ML-driven approaches, future research could explore multimodal data sources, such as textual, visual, and auditory inputs. However, it is crucial to note that the efficacy of these ML models hinges on the accuracy of feature values. When relying on self-reported questionnaires, there is potential for inaccuracies due to various factors, including mandatory appointments or the nature of certain mental conditions. Therefore, future research should focus on crafting techniques that can distinguish genuine responses from potentially misleading ones, further strengthening the reliability of the outcomes.

## Data Availability

The dataset used for this study is a publicly available dataset which can be found in https://github.com/dinisurunisal/Suicide-Risk-Prediction-Project/blob/master/DataScience/AlgorithmComparison/Test-Data-10.csv

## Code availability

The code for training and testing of machine learning models used in this study is available on GitHub: https://github.com/arekavandi/XAI_Suicide
